# Rationale, design, and methodology for the optimizing outcomes in women with gestational diabetes mellitus and their infants study

**DOI:** 10.1186/1471-2393-13-184

**Published:** 2013-10-10

**Authors:** Diane C Berry, Madeline Neal, Emily G Hall, Todd A Schwartz, Sarah Verbiest, Karen Bonuck, William Goodnight, Seth Brody, Karen F Dorman, Mary K Menard, Alison M Stuebe

**Affiliations:** 1 School of Nursing, The University of North Carolina at Chapel Hill, Campus Box 7460, Chapel Hill 27599-7460, NC, USA; 2 Department of Obstetrics and Gynecology, Division of Maternal-Fetal Medicine, University of North Carolina School of Medicine, 3010 Old Clinic Building, Campus Box 7516, Chapel Hill 27599-7516, NC, USA; 3 Department of Family and Social Medicine, Albert Einstein College of Medicine, Rochester 14602, NY, USA; 4 Department of Obstetrics, Gynecology & Women’s Health, 1300 Morris Park Avenue, Bronx 10461, NY, USA; 5 Rex Healthcare Inc, OB/GYN, 4420 Lake Boone Trail, Raleigh 27607, NC, USA; 6 WakeMed Health & Hospitals, WakeMed Faculty Physicians, OB/GYN, 3024 New Bern Avenue, Raleigh 27610, NC, USA; 7 Department of Maternal and Child Health, Gillings School of Global Public Health, The University of North Carolina at Chapel Hill, 170 Rosenau Hall, Campus Box 7400, Chapel Hill 27599-7400, NC, USA

## Abstract

**Background:**

Women who are diagnosed with gestational diabetes mellitus (GDM) are at increased risk for developing prediabetes and type 2 diabetes mellitus (T2DM). To date, there have been few interdisciplinary interventions that target predominantly ethnic minority low-income women diagnosed with GDM. This paper describes the rationale, design and methodology of a 2-year, randomized, controlled study being conducted in North Carolina.

**Methods/Design:**

Using a two-group, repeated measures, experimental design, we will test a 14- week intensive intervention on the benefits of breastfeeding, understanding gestational diabetes and risk of progression to prediabetes and T2DM, nutrition and exercise education, coping skills training, physical activity (Phase I), educational and motivational text messaging and 3 months of continued monthly contact (Phase II). A total of 100 African American, non-Hispanic white, and bilingual Hispanic women between 22–36 weeks of pregnancy who are diagnosed with GDM and their infants will be randomized to either the experimental group or the wait-listed control group. The first aim of the study is to determine the feasibility of the intervention. The second aim of study is to test the effects of the intervention on maternal outcomes from baseline (22–36 weeks pregnant) to 10 months postpartum. Primary maternal outcomes will include fasting blood glucose and weight (BMI) from baseline to 10 months postpartum. Secondary maternal outcomes will include clinical, adiposity, health behaviors and self-efficacy outcomes from baseline to 10 months postpartum. The third aim of the study is to quantify the effects of the intervention on infant feeding and growth. Infant outcomes will include weight status and breastfeeding from birth through 10 months of age. Data analysis will include general linear mixed-effects models. Safety endpoints include adverse event reporting.

**Discussion:**

Findings from this trial may lead to an effective intervention to assist women diagnosed with GDM to improve maternal glucose homeostasis and weight as well as stabilize infant growth trajectory, reducing the burden of metabolic disease across two generations.

**Trial registration:**

NCT01809431

## Background

Pregnancy is an unrealized window of opportunity for primary prevention of diabetes. Approximately a third of women diagnosed with type 2 diabetes (T2DM) have a history of gestational diabetes mellitus (GDM), defined as carbohydrate intolerance during pregnancy [1,2]. GDM affects 1 in 20 pregnant women in the U.S. [1,2]. These women are at increased risk of developing GDM in subsequent pregnancies and of developing T2DM later in life [1,3-5], with a cumulative incidence as high as 70% [6]. Infants born to women with GDM have a 2-fold risk of developing obesity [7] and a greater risk of developing T2DM than infants born to normoglycemic women [8,9]. There is strong evidence that lifestyle interventions can reduce the risk of progression to T2DM by 58% in the general population [6,10-12] and by 55% among women with a history of GDM [6]. The American College of Obstetricians and Gynecologists [13] and the American Diabetes Association [1,2] recommend that women with GDM be counseled about diet, exercise, and weight reduction. However, several studies suggest that the advice is insufficient to change maternal behavior [14-16]. Effective interventions are therefore needed to improve health-promoting behaviors among women with a history of GDM. One-on-one lifestyle interventions such as the Diabetes Prevention Program are costly and did not target pregnant women [17]. However, group interventions have been found to be both acceptable and effective in promoting positive pregnancy behaviors [18,19]. Studies of women with GDM have found self-efficacy to be one of the few modifiable predictors of physical activity [20-22]. A recent study demonstrated that breastfeeding may reduce risk of progression to metabolic syndrome by as much as 86% by utilizing from 400 to 600 calories a day and stabilizing blood sugar [23].

It is imperative that women diagnosed with GDM decrease their BMI to < 25 kg/m2 after the birth of their infant, both to improve metabolic outcomes and to decrease progression to T2DM later in life [1,2]. Among reproductive aged women, excessive gestational weight gain is a consistent predictor of worsening obesity [24,25]. A total of 40% of normal weight women and 60% of overweight women exceed the Institute of Medicine (IOM) prenatal weight gain guidelines [26,27]. Compared with women who gain within recommended ranges, women who gain excessively are more likely to be overweight or obese 10 or more years later [24,28].

These risks are magnified among women with GDM, because those who are overweight or obese and do not lose weight postpartum are at increased risk for developing prediabetes, T2DM, hypertension, hypercholesterolemia and cardiovascular disease later in life [1,2]. The estimated total economic cost of diagnosed diabetes in 2012 was $245 billion, a 41% increase from the previous estimate of $174 billion (in 2007 dollars) [29].

To reduce the risk of progression to T2DM, interventions must counter secular trends in diet and physical activity. Nutritional quality has declined with an increased intake of sugared beverages and high calorie and high fat foods [27,30,31]. Less than 50% of adults currently eat five to six servings of vegetables per day [30,32]. Fast food intake has increased along with increased caloric intake and increased weight in adults [33].

Physical activity has decreased and sedentary behavior has increased both among in adults in the general population and among pregnant women [34]. Current physical activity guidelines for prenatal and postpartum women is 30 minutes on most if not all days of the week [35]. Approximately 75% of pregnant women do not meet the recommended levels of daily physical activity [36].

The intervention to be tested in our trial includes a comprehensive group **N**utrition and **E**xercise Education, Coping **S**kills **T**raining (**NEST**), and exercise intervention [37-39]. During pregnancy, participants receive a prenatal session on the benefits of breastfeeding for metabolic control and infant health [40-46]. At 6 weeks postpartum, they receive a session on diabetes education, and messages are reinforced with weekly educational and motivational text messages from the time of enrollment to completion of participant’s time in the study.

### Theoretical framework for the intervention

The study intervention is based on social cognitive theory [47-50], which posits that learning and practicing a new behavior enhances self-efficacy. When self-efficacy is enhanced, the probability that the new behavior will be maintained increases [47,48,50]. When an individual cannot effectively solve a problem, their confidence for dealing with the next problem is decreased [47-50]. Coping skills training assists mothers in dealing with problems they encounter as they incorporate new nutrition and exercise behaviors [47-50]. Our hypothesis is that cognitive-affective processes [51] will improve in women who receive breastfeeding, diabetes, nutrition and exercise education, coping skills training, exercise, a home-based exercise program, and weekly educational and motivational text messages. We further hypothesize that these women will increase self-efficacy, improve their nutrition and exercise behaviors, and manage their weight. These behavior changes, in turn, will decrease their risk of progression to T2DM and improve their infants’ outcomes [37-39,47-50].

### Aims

The first aim of the study is to determine the feasibility of the intervention, including its acceptability, and further refine intervention materials and study procedures (recruitment, enrollment, intervention, retention, and data collection). The second aim of study is to test the effects of the intervention on maternal outcomes from baseline at 22–36 weeks pregnant (Time 1) to 6 weeks postpartum (Time 2), 4 months postpartum and completion of Phase I (Time 3), 7 months postpartum and completion of Phase II (Time 4), and 10 months postpartum, after 3 months on their own (Time 5). The primary maternal outcome is a decrease in fasting blood glucose and weight from baseline to 10 months postpartum. Secondary maternal outcomes include a decrease in glucose levels from the oral glucose tolerance test, insulin levels, homeostatic model assessment of insulin resistance (HOMA-IR), glycated hemoglobin A1C (A1C), lipid panel, blood pressure, and adiposity, as well as improvement in health behaviors, eating, exercise and breastfeeding self-efficacy from baseline to 10 months postpartum. The third aim of the study is to quantify the effects of the intervention on infant feeding and growth outcomes (weight status [weight-for-length]) and breastfeeding (weeks until stopped breastfeeding, weeks exclusively breastfed, and intensity of breastfeeding) from birth to 10 months postpartum.

## Methods

### Design

**Figure 1:**
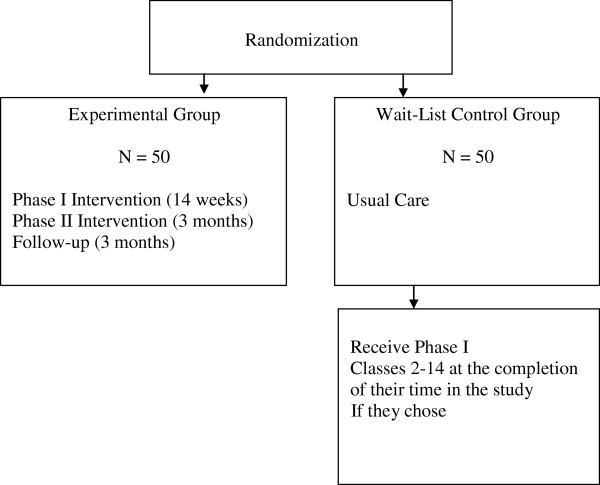
Randomization for the trial.

The study will use a two-group, randomized, repeated measures design to test the feasibility of the adapted NEST intervention for women diagnosed with GDM during their current pregnancy. See Figure 1. An experimental group of women (n = 50) will receive a two-phased intervention with follow-up. In Phase I (Intensive Intervention), interventionists will meet with women in small groups for 14 sessions. The initial session, during pregnancy, will focus on the benefits of and strategies to sustain breastfeeding. The remaining sessions, on diabetes, nutrition and exercise education, coping skills training, exercise, and a home-based exercise program, will start when they are about 6 weeks postpartum. Participants will also receive weekly educational and motivational text messages. In Phase II (Continued Support), interventionists will hold monthly meetings with the women for 3 months to discuss problems related to breastfeeding, nutrition and exercise, and to provide feedback and support. Data will be collected at baseline at 22–36 weeks pregnant (Time 1), to 6 weeks postpartum (Time 2), 4 months postpartum and completion of Phase I (Time 3), 7 months postpartum and completion of Phase II (Time 4), and 10 months postpartum, after 3 months on their own (Time 5). Time 3 and Time 4 data collections will determine the magnitude of intervention effects after Phase I and Phase II respectively; Time 5 will determine whether women have maintained their new skills. Three to 6 months after completion is a standard length of time for follow-up in weight management studies [52].

A wait-list control group (n = 50) will receive usual care and will have the same data collection schedule as the experimental group (Time 1-Time 5). After they complete the Time 5 data collection, they will be offered sessions 2–14 only.

### Settings

The study is being conducted in partnership with the University of North Carolina at Chapel Hill (UNC-CH) Schools of Nursing and Medicine and two clinical sites, Rex Healthcare and WakeMed. Our team has had a longstanding relationship with both sites. A local church near Rex Healthcare and a community center near WakeMed are being utilized for enrollment, data collection and delivery of the intervention. Women will be enrolled during four enrollment periods. After each of the four enrollment periods, the sites will be randomized to the experimental or wait-list control group. Women will be told their group assignment by phone.

### Power analysis

Eighty women are expected to complete the study; 40 per group. We will induct 100 women to take into account a 20% attrition rate seen in our previous studies [37-39,53]. While individually randomizing women to either the experimental or wait-list control group would be ideal, this is not feasible in our settings because of the limited number of women with GDM available in each site during any given enrollment period. Therefore, we have chosen a cluster-randomized approach. After a group of women is inducted, they will be randomly assigned to either the experimental group or the wait-list control group, to provide a balanced allocation in each site, yielding a random sequence of two experimental groups and two wait-list control groups for each site. This will provide two-group, longitudinal comparisons of complete data on 40 women per group, for analyses of changes in metabolic, clinical, weight, adiposity, health behaviors, and self-efficacy at up to four post-baseline time points. A cluster power calculation was performed with POWERLIB20 SAS/IML modules, which incorporate the methods described in Muller [54]. These methods calculate power for the general linear multivariate model, including repeated measures data, allowing adjustment for within-cluster correlation, which is expected to be small. We intend to determine effect size for each maternal and infant outcome addressed in Aims 2 and 3 in separate multivariate models. The study will be well powered to detect moderate to large effect sizes. An effect size of .67 SD at any post-baseline time point with this sample size will provide .80 power at the two-sided .05 significance level; an effect size of .8 SD increases the power to .92.

### Sample

The trial received ethical approval by the Institutional Review Board at The University of North Carolina at Chapel Hill, REX Healthcare and WakeMed. Women between 22–36 weeks of pregnancy who are diagnosed with GDM are eligible to enroll in the study. Inclusion criteria include a diagnosis of GDM during the current pregnancy by two or more 100 g Oral Glucose Tolerance Test values exceeding established thresholds (fasting 95, 1 h 180, 2 h 155, 3 h 140 mg/dL) [55]; age 18 years or older; a pre-pregnancy body mass index ≥ 25 kg/m^2^; ability to read and write in English; and willingness to consent for themselves and their infant. Health care providers will not be asked to give permission for women to join the study. Women will be excluded if they have an A1C ≥ 6.5 mg/dL indicating T2DM. The project manager contacted both sites and met with health care providers and staff to explain the study and distribute brochures. Any woman diagnosed with GDM who meets the inclusion criteria is given a brochure and asked to call the study number. The woman is then screened over the phone by the project manager. If the woman meets inclusion criteria and is interested in joining the study, an enrollment appointment is set up at one of the community centers at a convenient time for the woman to confirm eligibility and review the consent. The project manager confirms eligibility, explains the study, and answers all questions before asking the woman to consent for herself and her infant.

### Phase I intensive intervention

The Phase I classes are adapted from *Children and Parents Partnering Together to Manage their Weight*[56] and the feasibility study *Reducing the Risk of Chronic Disease: A Group- Based Weight Loss Program for Postpartum Mothers*[57] with additional sessions about 1) the importance of breastfeeding for metabolic control and infant health and 2) reinforcement of GDM reoccurrence with future pregnancies, and progression to prediabetes and T2DM [37-39,53]; and weekly educational and motivational text messages until women have completed their time in the study. Each 60-minute session will be delivered to a group of 12–13 women (Table 1). All women at both sites receive a GDM education class at the time of diagnosis by a certified diabetes educator. These existing classes are virtually identical. We have added two classes. We will deliver the first class during pregnancy, on the benefits of breastfeeding for metabolic control and for infant health. We will deliver the second class when women are 6 weeks postpartum to reinforce knowledge of recurrent GDM and progression to T2DM. To further support breastfeeding, our interventionists, who have experience in breastfeeding support, will contact experimental women weekly until they start classes (6 weeks postpartum) to inquire how breastfeeding is progressing and answer questions using a protocol we have developed with a consultant for our study, based on an effective pre- and post-natal breastfeeding intervention protocol [58]. We have a lactation consultant on the grant that will be available to assist with mother’s breastfeeding concerns. Also, at each site, registered dietitians will be available to discuss caloric needs with breastfeeding mothers in the experimental group [59].

**Table 1:** Phase I intensive intervention

**Pregnancy**	
Class 1	The benefits of breastfeeding for metabolic control and for your infant’s health
**Postpartum**	
Class 2	Reinforcing knowledge about recurrent GDM and progression to T2DM
Class 3	The importance of daily exercise
Class 4	Understanding calories, proteins, carbohydrates, and fats
Class 5	How portion control can make a difference
Class 6	How to make healthy substitutes with food
Class 7	Choosing healthy food when eating out
Class 8	Increasing exercise (cognitive restructuring)
Class 9	Improving nutrition and exercise behaviors (social problem solving)
Class 10	Motivating yourself in a positive manner (assertiveness training)
Class 11	Understanding barriers to healthy choices (social problem solving)
Class 12	Getting back on track after relapse (assertiveness training)
Class 13	Working through conflict (conflict resolution)
Class 14	Putting it all Together

Beginning with class 2, the women will also participate in a weekly exercise session for 60 minutes that will include a 5-minute warm up, 30 minutes of aerobic exercise, a 10-minute cool down, and 15 minutes to go over the home-based exercise program handout of the week. Classes will include group walking, zumba dancing, and cardio kickboxing, which have been well received in our previous interventions [56,57]. At the end of each session 2–14, women will be asked to set a nutrition and exercise goal for the next week. Women will also receive an educational or motivational text message weekly from the time of enrollment until completion of their time in the study. If a woman is unable to attend a session, the interventionist will call them and make up class content over the phone.

### Phase II continued support

The experimental group women will then return to the site for a group session once a month for 3 months. They will receive a reminder phone call several days before each session. Women will be weighed and have a group discussion, run by the interventionist, who will answer questions and help women problem solve issues around nutrition, exercise, and breastfeeding. If a woman misses a session, the interventionist will call and ask how she is doing. Content will not be made up. The women will continue to receive monthly educational and motivational text messages to maintain contact.

### Integrity of the intervention

Integrity of the intervention will be assessed by observation of two randomly selected sessions per month. The project manager will use a checklist to score sessions based on content identified in the intervention protocol. Drift will be defined as teaching less than 80% of the protocol content. If drift occurs, the interventionist will be retrained until the protocol is followed consistently. We will use a checklist of behavioral indicators to assess the intervener’s skills in facilitating sessions, engaging the women, problem solving, providing positive feedback, and goal setting. Retraining will be provided as necessary.

### Wait-list control group

Women in the wait-list control group will receive usual care. Data on the women and infants will be collected at the same times as on the women and infants in the experimental group, and women will receive $10 each time they complete a data collection visit. Wait-list control group women will receive reminder calls one week before each visit. Transportation vouchers and childcare will be provided. During the study, they will receive a monthly card to thank them for their continued participation. When they have completed the Time 5 data collection, they will be offered the Phase I intervention (sessions 2–14) classes.

### Measurement

Table 2 shows the measures to be used, data sources, and measurement times. All instruments have been evaluated for their psychometric qualities in previous studies [56,57] and will be reevaluated in this study. Data will be collected at baseline at 22–36 weeks pregnant (Time 1), 6 weeks postpartum (Time 2), 4 months postpartum and completion of Phase I (Time 3), 7 months postpartum and completion of Phase II (Time 4), and 10 months postpartum, after 3 months on their own (Time 5). Completion of data collection takes approximately 45 minutes for each mother.

**Table 2:** Summary of measures

**Variables and Their measurement**	**Respondent**	**T1**	**T2**	**T3**	**T4**	**T5**
**Aim 1: Feasibility**						
Process Evaluation Checklist	Project Manager		X	X	X	
Intervention Session Evaluation	Mothers	X	X	X	X	
Screening and Enrollment logs	Project Manager & Research Assistants	X				
Field Notes	Interventionists & Research Assistants	X	X	X	X	X
Intervention Attendance	Interventionists	X	X	X	X	
Data Collection Attendance	Research Assistants	X	X	X	X	X
Exit Interview	Mothers					X
**Aim 2: Maternal Outcomes**						
**Metabolic and Clinical Outcomes**						
3 hour Oral Glucose Tolerance Test ^1^ (FBG)	Mothers	X				
2 hour Oral Glucose Tolerance Test ^2^ (FBG)	Mothers		X			X
Insulin Level and HOMA-IR	Mothers	X	X			X
A1C and Lipid Panel	Mothers	X				X
Blood Pressure	Mothers	X	X	X	X	X
**Weight Status Outcomes**						
Height, Weight, Body Mass Index	Mothers	X	X	X	X	X
**Adiposity Outcomes**						
Waist Circumference	Mothers	*	X	X	X	X
Triceps & Subscapular Skinfolds	Mothers	X	X	X	X	X
**Health Behavior Outcomes**						
Adult Health Behavior Survey	Mothers	X	X	X	X	X
Health Promoting Lifestyle Profile II	Mothers	X	X	X	X	X
Accelerometry for 7 days	Mothers	X	X	X	X	X
**Self-Efficacy Outcomes**						
Eating Self-Efficacy Scale	Mothers	X	X	X	X	X
Exercise Self-Efficacy Scale	Mothers	X	X	X	X	X
Breastfeeding Self-Efficacy Scale	Mothers	X	X	X	X	X
**Aim: 3 Infant Feeding and Growth**						
Weeks until breastfeeding stopped ^#^	Mothers		X	X	X	X
Weeks exclusively breastfed ^#^	Mothers		X	X	X	X
Intensity of breastfeeding ^#^	Mothers		X	X	X	X
Weight-by-Length Z scores	Infants		X	X	X	X
**Demographic Data**	Mothers	X				

T1 (Baseline, at 22–36 weeks); T2 (6 weeks postpartum); T3 (4 months postpartum at completion of Phase I); T4 (7 months postpartum at completion of Phase II); T5 (10 months postpartum, after 3 months on their own); ^1^ 3 hour OGTT performed as part of GDM diagnosis includes fasting, 1, 2, and 3 hour glucose after a 100 g load; ^2^ 2 hour OGTT includes a fasting and 2 hour glucose after a 75 g glucose load; * Not done pregnant; ^#^ If a mother stops breastfeeding we will stop collecting data.

To assess acceptability of the intervention (Aim 1), the project manager will observe two randomly selected sessions per month to assess whether the sessions are being taught by the protocol. Participants will be asked to complete an evaluation at the completion of each session. At the Time 5 data collection, a trained research assistant will conduct a 15-minute exit interview with all experimental group women. To assess other aspects of feasibility, the project manager and research assistants will keep screening and enrollment logs on recruitment efforts; the number who were ineligible, with reasons for ineligibility; and the number who declined to participate with reasons why. The interventionists will keep field notes and collect data on attendance at each session, reasons for non-attendance, and number of make-up sessions provided. To assess the feasibility of the instruments, the research assistants will keep field notes and collect data on length of time it takes participants to complete each instrument, challenges encountered, and the ways they were resolved.

For maternal outcomes (Aim 2), research assistants blinded to the study group assignment will collect all data. The research assistants will use a standardized manual for collecting physiological and questionnaire data. Prior to each data collection, the research assistants are tested for inter-rater reliability on height, weight, waist circumference, triceps and subscapular skinfolds, and weight-for-length measurements.

### Metabolic and clinical outcomes

#### Oral Glucose Tolerance Test (OGTT)

The results of the mothers’ 3-hour OGTT will be copied from the medical record as Time 1 (baseline) data. The mother’s 2-hour OGTT will be scheduled through the outpatient laboratory and laboratory results will be received by secure fax from the laboratories. Women diagnosed with GDM will have two or more 100 g OGTT values exceeding established thresholds: (fasting 95, 1 h 180, 2 h 155, 3 h 140 mg/dL) [55]).

#### Insulin levels

Insulin will be measured from a fasting sample of venous blood. Aliquots of plasma will be separated, labeled and stored at −80 degrees Celsius. Insulin testing will be completed at Rex Healthcare and WakeMed laboratories. Both laboratories use the same radioimmunoassay kit, which has less than 1% cross-reactivity with C-peptide and proinsulin. The inter-assay variation for insulin is 12% and intra-assay cross-reactivity is 11%, which is well within acceptable levels. These assessments will allow us to confirm the validity of the HOMA- IR. Normal values are 5–20 micro units per milliliter while fasting [1,2].

#### HOMA-IR

The HOMA-IR estimates the steady state of beta cell functioning and insulin sensitivity [60]. HOMA-IR has been shown to discriminate across race and ethnic groups and provides a good approximation of more complex tests [60]. Fasting glucose and insulin will be drawn on all study participants by experienced laboratory personnel at either Rex Healthcare or WakeMed. The formula to be used is: (fasting insulin (IU/ml) × fasting glucose (mmol/l)/22.5 [60]. Greater HOMA-IR values indicate reduced insulin sensitivity. A value over 2.2 is indicative of insulin resistance [60].

#### Glycated hemoglobin (A1C)

A1C is measured using an immunoassay that measures the concentration of a macromolecule in a solution through the use of an immunoglobulin. A1C will be drawn on all study participants by experienced laboratory personnel at either Rex Healthcare or Wake Med. An A1C ≥ 6.5 will be considered a diagnosis of type 2 diabetes [1,2]. and those women will be excluded from the study and referred to an endocrinologist.

#### Lipid panel

Total cholesterol, low-density lipoprotein (LDL), high-density lipoprotein (HDL) and triglycerides will be drawn and run by experienced laboratory personnel at either Rex Healthcare or WakeMed. Lipids will be determined by automated methods using the Hitachi 704 Analyzer (Roche Diagnostics). Desirable total cholesterol levels are below 200 mg/dL in adults. Normal fasting triglyceride levels are below 150 mg/dL. Desirable LDL levels are below 130 mg/dL. A low HDL is considered to be a value below 35 mg/dL, and high HDL, ≥60 mg/dL [61].

#### Blood pressure

Blood pressure will be measured with a Welch Alyn VSM 300 automatic blood pressure machine (Welch Alyn). The cuff bladder will cover at least two-thirds of the upper right arm and at least half of the circumference. The participant will sit quietly for 10 minutes prior to measurement. Blood pressure will be taken twice with at least 5 minutes between measurements, using recommended procedures [62,63].

### Weight status outcomes

Height will be measured twice and averaged on all women in street clothes without shoes. A portable stadiometer will be calibrated to 1/8-centimeter intervals. Weight will be measured twice and averaged in a private room on all women in street clothes and without shoes using a Tanita WB-110A Digital Scale. Body mass index (BMI) of mothers will be calculated twice by entering height and weight (kg/m^2^) [64,65]. In adults, overweight will be defined as a BMI between 25.0 to 29.9 kg/m^2^ and obesity will be defined as above 30.0 kg/m^2^[64,65].

### Adiposity outcomes

All adiposity measures will be taken in a private room by two research assistants. All adiposity measurements will be taken three times and averaged according to the National Health and Nutrition Examination Survey procedures [64,65]. Waist circumference will be measured using a Figure Finder measuring tape with lock (Novel Products Inc., Rockton, IL). Triceps and subscapular skinfolds will be measured using Lange skinfold calipers.

### Health behavior outcomes

The Adult Health Behavior Survey [66] will be used to collect information on intake of fruits, vegetables, sugared beverages, water, and fast food. Responses on the 23-item questionnaire will be recorded a 1 for a healthy behavior and 2 for an unhealthy behavior. Alpha coefficients in adults have ranged from 0.80 to 0.86 [66].

The Health Promoting Lifestyle Profile II (HPLP II) will be used to measure health promoting behaviors [67]. The HPLP II is a 48-item questionnaire that uses a 4-point Likert scale with responses including never, sometimes, often or routinely. The four subscales used for this study include health responsibility, exercise, nutrition, and stress management. Alpha coefficients have ranged from 0.78 to 0.93 for the subscales [67].

Accelerometry will be measured for 7 continuous days using the Computer Sciences and Applications (CSA) uniaxial accelerometer to capture weekday and weekend physical activity. Accelerometers do not rely on self-report and provide an objective measure of physical activity [68,69]. Information from the use of accelerometry assesses the intensity, frequency, and duration of activity performed [68]. Taking multiple days of CSA measurements and averaging them has been reported to increase alpha coefficients from 0.42-0.47 for 1 day to 0.7 for 4 days in adults [69,70]. The research assistant will explain the accelerometer to the mother and review the procedure. The mother will be provided with a log book to keep track of her daily activity and a phone number to call if she needs help. Mothers will be provided with a mailer to mail the accelerometer back at the end of the 7 days. A research assistant will send out a text message to the mother in the morning to remind her to put her accelerometer on.

### Self-efficacy outcomes

The Eating Self-Efficacy Scale [71] will be used to measure dietary self-efficacy. The questionnaire consists of 25-tems and will ask women to rate their difficulty in controlling eating on two subscales, which include the negative affect scale and the socially acceptable scale with a 1 reflecting no difficulty to a 7 reflecting a high degree of difficulty. Socially acceptable eating is defined as overeating at holiday or family events. Negative affect eating is defined as emotional eating. Alpha coefficients have been found to be 0.85 for the socially acceptable eating subscale and 0.94 for the negative affect subscale [71].

Breastfeeding Self-Efficacy will be measured using the Breast Feeding Self-Efficacy Scale [72]. The 14-item questionnaire will ask women how sure they are regarding breastfeeding with a 1 reflecting not at all confident to a 5 reflecting very confident. Alpha coefficients range from .92 to .96 [72].

### Infant outcomes

For infant feeding and growth (Aim 3), we will measure breastfeeding duration and intensity as a modifiable risk factor for maternal and infant metabolic disease. We will define breastfeeding duration as the time since birth, in weeks, until the mother stops breastfeeding or expressing milk for her infant. We will define exclusive breastfeeding as the time, in weeks, until the mother introduces formula or complementary foods. We will define breastfeeding intensity as the percent of all milk feeds that are breast milk (# of breast milk feeds per day) / (# of breast milk + formula + other milk feeds per day) [73,74]. We will measure weight-for-length in infants, using standardized weight-for-length z scores [75]. Length will be measured twice and averaged using a portable length board and weight will be measured twice and averaged using a portable calibrated digital scale according to procedures.

### Demographic data

Women will complete a demographic form, including questions about age, race/ethnicity, marital status, and income level.

### Procedures

The project manager will call women who have shown an interest in the study, give a verbal description of the study and ask pre-pregnancy height and weight. If they meet inclusion criteria, the research assistant will schedule an appointment and meet the woman at the site. The research assistant will give the woman an oral description of the study, requirements of participants, and the risks and benefits of participating; random assignment will be explained; and all questions will be answered. After informed consent, physiological data will be collected in a private room at the site. The research assistant will collect data in the same order each time: height, weight, waist circumference, triceps and subscapular skinfolds, and questionnaires, which should take 45 minutes. The 3-hr OGTT at Time 1 will be abstracted from the medical record. Data on infant length-by-weight will be collected following standard procedures.

### Data management

Participants will be tracked using ID numbers. All data will be double entered by different research assistants into a SAS database and backed up on a secure central computer network system. Comparisons will be run for the two versions, and inconsistencies will be checked against the raw data and corrected. Data will undergo range, consistency, and outlier checks. An audit trail will be established to document any changes.

### Data analysis

#### Aim 1

To determine the acceptability of the intervention sessions and materials, the investigators will analyze the interventionists’ field notes and exit interviews with women, which will be tape-recorded with participants’ permission, transcribed, and analyzed for themes. The findings will be used to further adapt and refine the intervention*.* To assess other aspects of feasibility, recruitment yield will be described by the number contacted and the number screened required to yield one enrollment. Interventionists will keep attendance records for each class. Retention will be described at each time point. Statistics on data collection, such as duration of collection per woman, will also be computed at each time point. Rates of recruited, screened, and retained women will be presented by group and time point, along with 95% confidence intervals; the upper bounds will provide an estimate of worst case values for rates in a future study.

#### Aims 2 and 3

To analyze maternal outcomes, descriptive statistics will be calculated for each measure at each time point. All analyses will be conducted using an intention-to-treat approach. Standardized effect sizes for each measure will be calculated for each time point as the ratio of the mean difference between the intervention and wait-list control group to its standard deviation. Correlation matrices will be computed to describe the degree of correlation of each measure across the repeated measures for each group. For the assessment of trends in Aim 2, preliminary analyses will be conducted to determine whether, despite randomization, the intervention and control groups were unbalanced at baseline on any measured variable. Any such variable will be examined for statistically significant relationships with any of the outcome measures, and if any are found, the variable will be considered as a covariate for subsequent models.

For each outcome mentioned in **Aims 2 and 3**, a separate linear mixed-effects model will be constructed. These will model data across all available post-baseline time points; contrasts will be constructed to evaluate the effects of the intervention over the post-baseline period, as compared to the wait-list control group, with covariate adjustment for baseline values. These models will include assessment for main effects for experimental group, time point, and site, as well as interactions, and will account for the within-site and within-subject correlation structure invoked via the study design. This approach will provide useful information in guiding the analysis of a future study. Least-squares means will be computed and plotted for each time point for each group to determine average trajectories for each measure.

## Discussion

Optimizing Outcomes in Women with Gestational Diabetes Mellitus and their Infants study will provide insight into the initial efficacy of a 14-week intervention that focuses on the benefits of breastfeeding, understanding GDM and progression to T2DM, nutrition and exercise education, coping skills training, physical activity, educational and motivational text messaging and 3 months of continued support. The results of the study will provide crucial information on feasibility of delivering the intervention in a hard to reach population. The extensive measurements on maternal outcomes including metabolic and clinical, weight, adiposity, health behavior and self-efficacy and infant feeding and growth outcomes will provide a strong foundation to develop an efficacy study. This trial may lead to an effective intervention to assist women diagnosed with GDM to improve glucose and weight as well as stabilize infant growth trajectory, thereby reducing the burden of metabolic decrease across two generations.

## Competing interests

The authors declare that they have no competing interests.

## Authors’ contributions

DCB and AMS are the multiple principal investigators and TAS and SV are co-investigators of the study. MKM is a senior research advisor, KB is a consultant and KFD is a research nurse. MN is the project manager and EGH is an interventionist. WG and SB are onsite study OB/GYN physicians. DCB, AMS, TAS, SV, MKM and KB contributed to developing the research questions and study design. DCB, MN, EGH, TAS, SV, KB, WG, SB, KFD, MKM, and AMS are contributing equally to implementation of the study protocol. All authors contributed in the development, read and approved the final manuscript.

## Pre-publication history

The pre-publication history for this paper can be accessed here:

http://www.biomedcentral.com/1471-2393/13/184/prepub
